# Xenogeneic platelet-rich plasma lotion for preventing acute radiation dermatitis in patients with breast cancer undergoing radiotherapy: An open-label, randomized controlled trial

**DOI:** 10.1016/j.jpra.2024.11.017

**Published:** 2024-12-04

**Authors:** Shin-Ting Chen, Guo-Shiou Liao, Chin-Jui Wu, Mao-Sen Cheng, Po-Chien Shen, Yu-Fu Su, Wen-Yu Chuang, Chia-Ni Lin, Kuen-Tze Lin, Chun-Shu Lin

**Affiliations:** a Department of Radiation Oncology, Tri-Service General Hospital, National Defense Medical Center, Taipei, Taiwan; b Division of General Surgery, Department of Surgery, Tri-Service General Hospital, National Defense Medical Center, Taipei, Taiwan; c School of Medicine, University of Edinburgh, Scotland, UK; d School of Public Health, National Defense Medical Center, Taipei, Taiwan; e Department of Biomedical Imaging and Radiological Sciences, National Yang Ming Chiao Tung University, Taipei, Taiwan; f School of Dentistry, China Medical School, Taichung, Taiwan; g Department of Radiation Oncology, Cardinal Tien Hospital, New Taipei City, Taiwan; h School of Medicine, College of Medicine, Fu-Jen Catholic University, New Taipei City, Taiwan

**Keywords:** PRP lotion, Breast cancer, Acute radiation dermatitis, Quality of life, Patient-reported outcome

## Abstract

**Background:**

Breast cancer patients experience acute radiation dermatitis (ARD) during radiation therapy (RT). This study investigated the prophylactic effect of a newly developed xenogeneic platelet-rich plasma (PRP) lotion on ARD for breast cancer patients.

**Methods:**

This study enrolled patients with ductal carcinoma in situ and early-stage invasive breast cancers after breast-conserving surgery. Hypofractionated whole-breast RT (42.5 Gy in 16 fractions) followed by tumour bed boost (10 Gy in 5 fractions) was used. The patients were randomly assigned to XONRID® gel (*n* = 48) or PRP lotion (*n* = 52) groups. We recorded the skin toxicity weekly during RT and at 2 weeks after RT. ARD was graded on the basis of the RTOG definition by two senior radiation oncologists, and the numerical rating scale (NRS) for pain and Dermatology Life Quality Index (DLQI) were subjectively scored by patients.

**Results:**

Grade 3–4 ARD was noted in three (6 %) patients in the XONRID® gel group and no patients in the PRP lotion group (*p* < 0.001). One patient did not complete RT in the XONRID® gel group due to intolerable pain and refused to complete the weekly questionnaires and follow-ups. Compared with the XONRID® gel group, the PRP lotion group had significantly reduced and delayed progression of mean ARD (*p* = 0.001), lower mean NRS for pain value (*p* = 0.021) and lower mean DLQI (*p* = 0.048).

**Conclusions:**

This randomized controlled trial is the first to use xenogeneic PRP lotion for ARD prevention. This lotion has prophylactic effects against ARD and thus improves quality of life of patients undergoing RT.

## Introduction

According to the 2022 Cancer Registry Annual Report by Taiwan's Health Promotion Administration, breast cancer has the highest incidence rate in Taiwan (18.18 %). The widespread use of mammography and ultrasound in Taiwan have enabled most breast cancers to be diagnosed at an early stage. Whole-breast radiation therapy (RT) with or without a tumour bed boost is the standard treatment for reducing local recurrence of early-stage breast cancer after breast-conserving surgery.^1^ During the RT course, patients often experience acute radiation dermatitis (ARD), a form of topical inflammation, which causes decreased sweating; pruritus; topical erythema; hyperpigmentation; dry and moist desquamation; pain and a burning sensation; and in the worst cases, necrosis, ulcer, and bleeding. ARD usually occurs in weeks 2–4 of RT and persists up to 4 weeks after treatment completion.^2^^,^^3^ Several parameters affect ARD severity and timing, including fraction size, total dose, beam quality, bolus, field size and depth, and concurrent use of systemic therapy. Advancements in RT techniques, such as the development of intensity-modulated RT, have resulted in significantly reduced incidence of moist desquamation.^4^ However, most patients continue to experience topical pruritus, erythematous skin reaction, and dry and moist desquamation.

In the last decade, several methods were developed for ARD prevention, including the use of topical moisturizing lotion or gel, barrier film, silicone-based film-forming gel dressing, topical biological preparations (e.g. epidermal growth factor, interleukins, plasma, and stem cells), and dermaprazole.^5^, ^6^, ^7^, ^8^ However, most of these methods provide hydration or a physical barrier rather than directly contributing to skin repair. Platelet-rich plasma (PRP) is a rich source of growth factors, such as platelet-derived growth factor, epithelial growth factor, and vascular endothelial growth factor. Several xenogeneic PRP products are commercially available for skin care in many countries and regions, including Taiwan, Japan, Korea, Thailand, and Europe. In Taiwan, xenogeneic PRP products have been widely used after cosmetic laser treatment in aesthetic medicine clinics for skin care and repair on the irradiated skin. In the present study, we investigated the prophylactic effect of xenogeneic PRP lotion on ARD in patients with breast cancer.

## Materials and methods

### Study design

This open-label randomized prospective study was approved by the Institutional Review Board of Tri-Service General Hospital (TSGHIRB No: C202105173, C202205004, A202205103 and A202305034) and followed the recommendations of the Declaration of Helsinki. All patients provided written informed consent before RT began.

We included patients in whom ductal carcinoma in situ and stage I–II breast cancers (invasive ductal carcinoma and invasive lobular carcinoma) without nodal metastasis were detected upon screening after breast-conserving surgery. All patients underwent RT; however, if a patient required systemic chemotherapy, it was administered first and followed with RT. We excluded patients with the following conditions: Eastern Cooperative Oncology Group performance status > 2, a gross residual tumour, inflammatory breast cancer, pathologic T4 category with skin invasion, a history of RT over the ipsilateral breast or chest wall, topical cellulitis in the treated area, poor surgical wound healing, pregnancy, generalized skin disease (such as Sjogren syndrome, psoriasis, or systemic lupus erythematosus), or nodal risk (including pathologic T2 with central or medial tumour location). The use of targeted therapy (e.g., trastuzumab or pertuzumab) or endocrine therapy (tamoxifen or aromatase inhibitors) was permitted during the RT course.

### Radiotherapy

All patients underwent hypofractionated whole-breast RT (42.5 Gy in 16 fractions) followed by a tumour bed boost (10 Gy in 5 fractions). The clinical target volume of the whole breast and tumour bed was with an additional 0.8 cm margin to ensure adequate coverage and account for potential patient movement or setup variations. The supraclavicular, infraclavicular, internal mammary, and axillary nodal regions were not present in the treatment field. We did not apply bolus over the breast to enhance skin dose, and there is no deliberate avoidance of wrinkles on the skin. Volumetric-modulated arc therapy was the most commonly used technique in our institute; however, intensity-modulated RT, traditional tangential opposed field technique, and tangential opposed field-in-field technique were also used. The treatment technique was selected at the discretion of the radiation oncologist and physicist responsible for treatment planning. However, in the treatment plan, we did not specifically contour the skin for avoidance in all patients.

### Skin care

Patients were randomly assigned to XONRID® gel group or PRP lotion groups. XONRID® topical gel is a water-based gel that forms a protective film. This gel reduces trans-epidermal water loss and enhances moisturization to prevent ARD. XONRID® gel is a valid topical dressing in the prevention of ARD in breast cancer patients, delaying time to develop skin toxicity and reducing the proportion of patient who experienced ≧grade 2 ARD during RT treatment and 2 weeks later.^9^ The PRP lotion (KimClaire, Taiwan) used in this study is a topical water-based lotion. Its main effective ingredient is xenogeneic PRP, which is derived from the blood obtained from Holstein cows raised in Taitung, Taiwan, without animal sacrifice. It was demonstrated to enhance the production of human type I procollagen in human Hs68 cell lines without cell toxicity by the Industrial Technology Research Institute, Taiwan, a third-party laboratory. Because PRP lotion is classified as a general cosmetic product, it is exempt from review by the Taiwan Ministry of Health and welfare. The patients in the PRP lotion group were instructed to apply the lotion twice a day, starting from the first day of RT; that is, to apply it once within 1 hour after the daily RT session and once again before bedtime, although not within 6 h before the next RT session. They were asked to continue applying the lotion on the weekends (when they did not receive RT). After completing the full course of RT, all the patients continued to use the lotions for two weeks to account for the possibility of delayed onset of high-grade dermatitis.

All patients were instructed regarding mild bathing at the irradiated site in accordance with the Multinational Association of Supportive Care in Cancer (MASCC) recommendation. They were also instructed to avoid the following potentially skin damaging behaviors during the study period: topical friction and massage, sun exposure, shaving of the regional axilla, and hyperthermia. Topical steroids, topical antibiotics, and other medications could be administered to treat ARD as determined by the attending physician.

### Evaluation of skin toxicity

Acute skin toxicity was scored and documented by radiation oncologists objectively (clinician-reported outcomes [CROs]) and by the patients themselves subjectively (patient-reported outcomes [PROs]) every week over the 4-week RT course and at 2 weeks after treatment completion (a total of five evaluations). For the CROs, the ARD level was reported using the Radiation Therapy Oncology Group criteria: grade 0, no change; grade 1, follicular, faint, or dull erythema or dry desquamation; grade 2, tender or bright erythema or patchy moist desquamation; grade 3, confluent, moist desquamation other than skin folds; and grade 4, ulceration, hemorrhage, or necrosis.^10^ We took a digital photograph of the RT field at every visit, and the severity of ARD was evaluated and discussed by two senior radiation oncologists.

For the PROs, the patients were asked to report their topical pain feeling and topical skin discomfort by using the numerical rating scale (NRS) and Dermatology Life Quality Index (DLQI) respectively. The 11-point NRS, with endpoints ranging from 0 to 10, is a simple, practical method for quantifying pain sensation.^11^ The DLQI is a short, straightforward 10-item questionnaire that can be completed within 3 min in an outpatient department.^12^ Each item is scored from 0 to 3, with total scores ranging from 0 to 30. The 10 items of the DLQI can be grouped under the following six headings: symptoms/feelings, daily activities, leisure, work, personal relationships, and treatment. The DLQI has been widely used for assessing skin problems and was validated for routine clinical use.^13^ For both the DLQI and NRS, the higher the scores, the more uncomfortable the area within the RT field was.

### Statistical analysis

**Figure 1:**
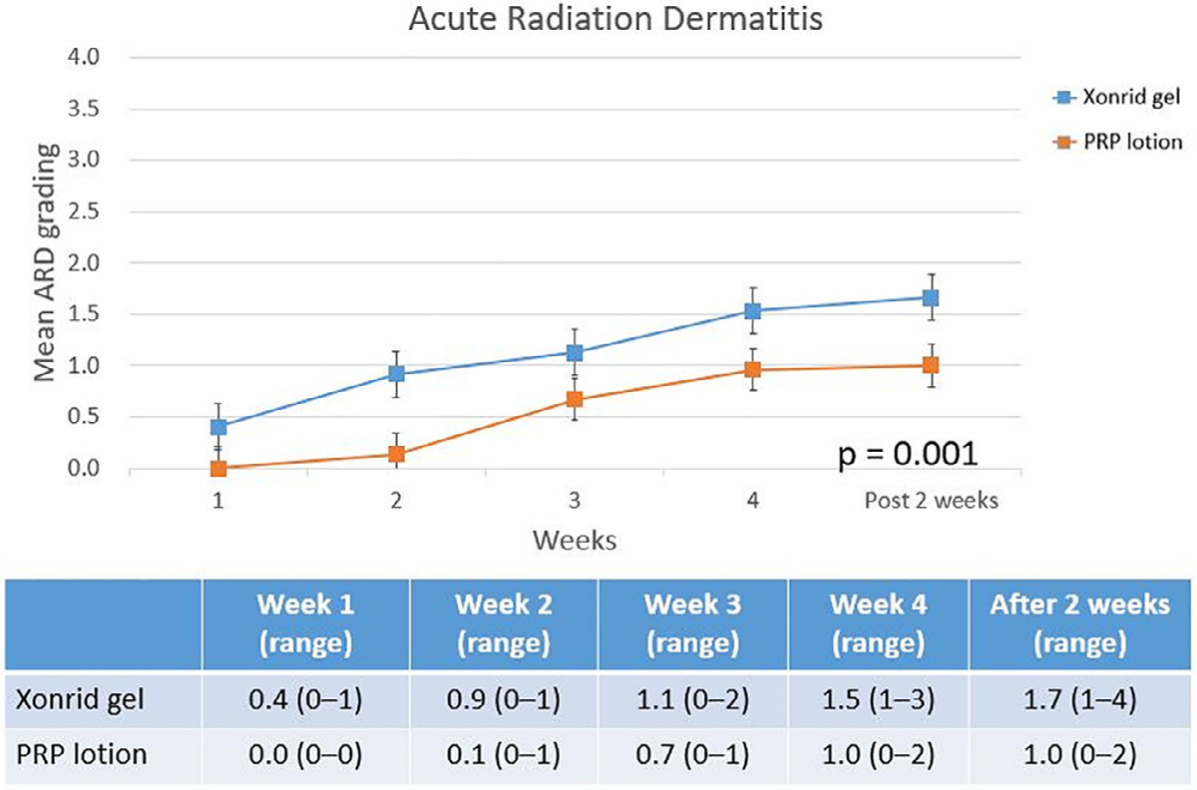
Acute radiation dermatitis grades (RTOG criteria) at five evaluation points: weekly averages during the 4-week RT and 2 weeks post-treatment. The PRP lotion group showed delayed onset and lower severity of ARD compared to the XONRID® gel group.

**Figure 2:**
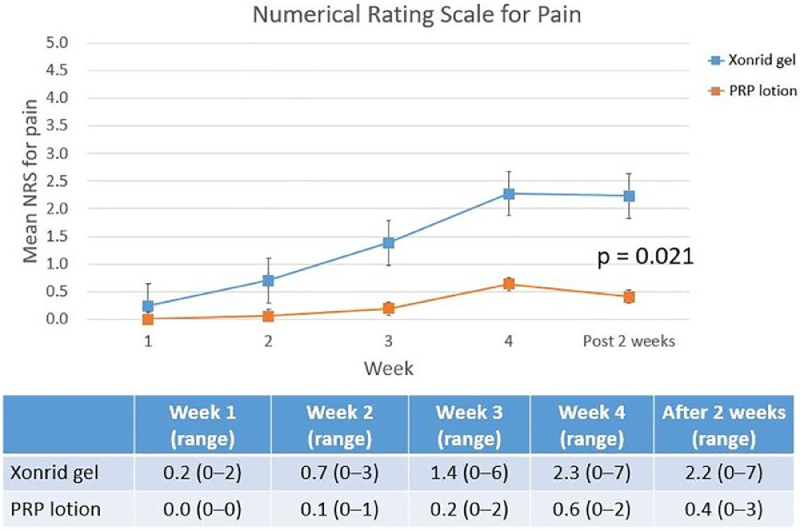
Numerical rating scale (NRS) scores for pain at five evaluation points: weekly averages during the 4-week RT and 2 weeks post-treatment. The PRP lotion group reported consistently lower pain levels than the XONRID® gel group from week 2 onward.

**Figure 3:**
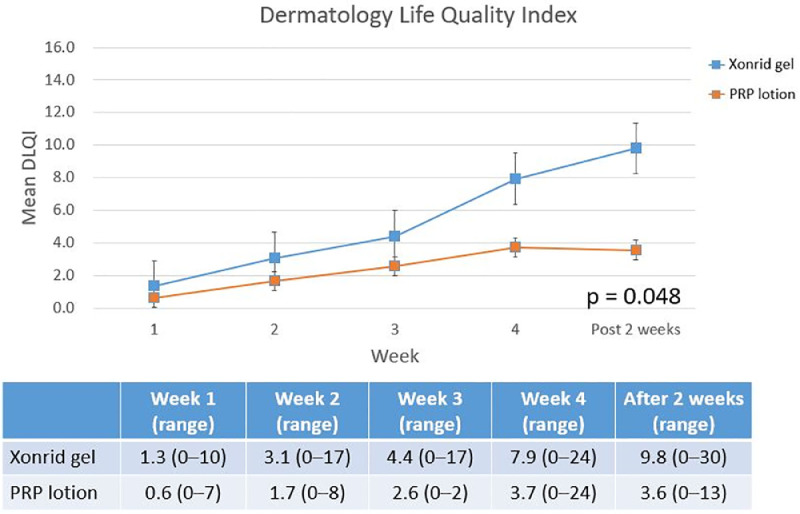
Dermatology Life Quality Index (DLQI) scores at five evaluation points: weekly averages during the 4-week RT and 2 weeks post-treatment. The PRP lotion group exhibited significant improvements in quality of life compared to the XONRID® gel group starting from week 4.

We used the Pearson chi-square or *t*-tests to compare the clinicodemographic factors between the two groups. The data are presented as medians and frequencies or percentages. All data were analyzed using an intention-to-treat analysis. The data in the Figure 1, Figure 2, Figure 3 represent weekly mean scores. SPSS version 20.0 (SPSS, Chicago, IL, USA) was used for all statistical analyses. All tests were two-sided, and *p* < 0.05 was set as statistically significant.

## Results

**Figure 4:**
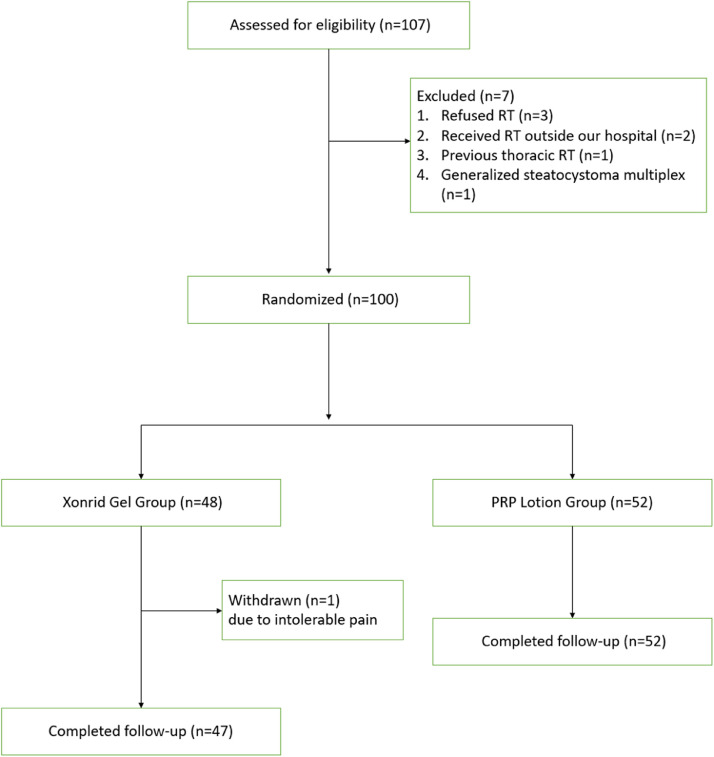
Flow diagram for the randomized controlled trial comparing xenogeneic PRP lotion and Xonrid® gel in preventing acute radiation dermatitis. Of 107 screened patients, 100 were randomized (PRP lotion: *n* = 52; Xonrid® gel: *n* = 48). One patient in the Xonrid® gel group discontinued due to pain, leaving 47 for analysis, while all 52 in the PRP lotion group completed the study. Randomization was done using a computer-generated block method with allocation concealment, and outcome assessors were blinded.

Between October 2021 and December 2023, 107 patients were screened after breast-conserving surgery. Seven patients were excluded (three refused RT, two received RT outside our hospital, one had previous thoracic RT, and one had generalized steatocystoma multiplex). Finally, 100 patients were included and randomized into the XONRID® gel (*n* = 48) or PRP lotion (*n* = 52) group. The patient and treatment characteristics are summarized in Table 1 and the flow diagram was shown in Fig. 4. The patients’ median age was 56 years (range, 25–85 years). Twenty-six patients were given a diagnosis of ductal carcinoma in situ. Twenty-one patients received adjuvant chemotherapy before RT. Five patients received targeted therapy concurrent with RT, and 33 received endocrine therapy concurrent with RT. The patient characteristics in the two groups were comparable. ARD in breast cancer patients primarily occurs in the skin folds of the breast area. These skin folds are mainly influenced by factors such as breast size, age, and BMI. According to the baseline data in our study (as shown in Table 1), there was no significant difference in age and BMI between the two groups. Moreover, the study participants were Taiwanese women, who inherently do not have significant differences in breast size, and the occurrence of extreme values (particularly large or small) is minimal. Therefore, there was no significant difference in skin folds between the two groups.

**Table 1:** Demographic and systemic treatment characteristics of patients in the XONRID gel and PRP lotion group.

Characteristics		Xonrid gel (*n* = 48)	PRP lotion (*n* = 52)	*p-*value
*Age (years)*	Median (range)	56 (25–83)	57 (37–85)	0.226
*BMI (kg/m2)*	Median (range)	22.7 (17–35)	24.4 (19–34)	0.063
*Stage TNM VIII Edition*	DCIS	10 (21 %)	16 (31 %)	0.234
	I	37 (77 %)	42 (61 %)	
	II	1 (2 %)	4 (8 %)	
*Previous chemotherapy*	no	39 (81 %)	40 (77 %)	0.633
	yes	9 (19 %)	12 (23 %)	
*Concurrent with targeted therapy*	no	46 (96 %)	49 (94 %)	0.731
	yes	2 (4 %)	3 (6 %)	
*Concurrent with endocrine therapy*	no	31 (65 %)	36 (69 %)	0.728
	yes	17 (35 %)	16 (31 %)	

Abbreviations: PRP, platelet-rich plasma; DCIS, ductal carcinoma in situ.

Table 2 shows the distribution of the most severe ARD grades across the two groups during the five evaluation visits. Grade 3–4 ARD was observed only in the XONRID® gel group (3/48, 6 %). In this group, 16 patients (33 %) had grade 1 ARD and 28 patients (59 %) had grade 2 ARD. In contrast, nearly all patients in the PRP lotion group (50/52, 96 %) experienced only grade 1 ARD. One patient in the XONRID® gel group discontinued RT due to intolerable pain and did not complete follow-up, leaving 99 patients for analysis. Figure 1, Figure 2, Figure 3 reflect the data from these 99 patients at all five evaluation points.

**Table 2:** Distribution of the most severe acute radiation dermatitis (ARD) grades in the XONRID gel and PRP lotion groups across all evaluation points.

	Xonrid gel (*n* = 48)	PRP lotion (*n* = 52)	*p*-value
*Grade 1*	16 (33 %)	50 (96 %)	<0.001
*Grade 2*	28 (59 %)	2 (4 %)	
*Grade 3*	2 (4 %)	0(0 %)	
*Grade 4*	1 (2 %)	0(0 %)	
*Incomplete RT due to pain*	1 (2 %)	—	

Abbreviations: PRP, platelet-rich plasma.

Fig. 1 shows that ARD onset occurred significantly later in the PRP lotion group compared to the XONRID® gel group (*p* = 0.001). There was no significant difference at week 1, but from week 2 onwards, the PRP lotion group consistently showed lower mean ARD grades, a difference that lasted until 2 weeks post-RT. Fig. 2 displays the mean NRS scores for pain, with the PRP lotion group reporting significantly lower scores than the XONRID® gel group (*p* = 0.021) from week 2 onwards. Fig. 3 illustrates the mean DLQI scores, with significant improvements in the PRP lotion group compared to the XONRID® gel group starting from week 4 (*p* = 0.048), although no significant differences were observed during the first 3 weeks.

## Discussion

According to the existing literature, this randomized controlled trial is the first to evaluate the effects of a xenogeneic PRP lotion on ARD prevention. The PRP lotion has superior effects to XONRID® gel with respect to preventing, delaying, reducing ARD severity and improving quality of life.

Recently, the MASCC published updated clinical practice guidelines for ARD prevention and treatment based on empirical research through a combined literature review and Delphi process; only Mepitel film and photobiomodulation therapy (PBMT) were recommended for the prevention of ARD on breast cancer. Most interventions could not be recommended by the MASCC due to a low quality of evidence or conflicting finding across multiple trials.^14^ However, due to the hot and humid climate in Taiwan, some patients experienced discomfort with Mepitel Film, limiting its use in routine practice. PBMT has also shown promising results in preventing grade 2 or higher ARD and improving patients' quality of life during radiotherapy. StrataXRT®, another silicone-based film-forming gel, has demonstrated comparable efficacy to Mepitel Film.^6^ Compared to other methods for preventing ARD, PRP lotion not only has preventive functions but also features skin repair and promotes growth factor proliferation. However, large-scale studies are still needed to compare their efficacy and benefits, including potential side effects, patient convenience, cost-effectiveness, and impact on healthcare resources.

PRP lotion's multiple mechanisms, such as promoting skin repair and stimulating growth factor proliferation, suggest its potential for broader clinical applications beyond breast cancer. As ARD is a common side effect in radiotherapy for various cancers, PRP lotion could provide protection in other cancer types. Moreover, PRP lotion might offer significant advantages for high-risk patients, such as those receiving high-dose radiotherapy or those with particularly sensitive skin. Therefore, future studies should further explore PRP lotion's efficacy across different cancer types and patient populations.

This study has some limitations. First, many patients reported that the most severe ARD occurred 3–7 days after RT completion. However, the last follow-up time was 2 weeks after RT completion, and we may have missed the most severe ARD in both groups. Second, the patients practiced skin care and PRP lotion application by themselves at home, and therefore, we could not assess their compliance. Third, the small sample size, especially that of patients with grade 3–4 ARD (*n* = 3) limited the statistical power of this study.

Future research should also consider different dosages and application methods to determine how these factors affect the efficacy of ARD prevention. For example, it remains unclear whether the frequency or amount of PRP lotion used influences its effectiveness. Further studies should also focus on improving PRP lotion's formulation to enhance its stability and skin absorption. Additionally, investigating the combined use of PRP lotion with other skincare products, such as protective films or gel-based products, may help maximize its preventive benefits. In clinical practice, integrating PRP lotion into routine radiotherapy care poses some challenges. One of the main issues is patient compliance, as self-application at home may lead to inconsistencies in usage, which could affect outcomes. Moreover, the cost-effectiveness of PRP lotion needs to be evaluated, especially for long-term use. Factors such as resource allocation in hospitals, the financial burden on patients, and product availability may influence its widespread adoption. Therefore, future studies should also address how to reduce costs and improve the ease of use to facilitate its broader clinical application.

The strengths of this study include the use of several subjective and objective methods to assess skin toxicity. Although the CROs and PROs of this study generally demonstrated strong concordance, the data presented in Figure 1, Figure 2, Figure 3 of our study reveal mild discrepancies. However, the PRP lotion group had more favorable CROs and PROs. The use of both CROs and PROs has become increasingly prevalent in clinical trials. Moreover, a study reported that patient distress during breast RT may be underrecognized.^15^ Future studies should develop tools to more accurately measure ARD by using CROs and PROs to thus improve data collection and improve the understanding of patients’ perceptions of their skin reactions, which can lead to improved patient care and reduce the likelihood of patients discontinuing RT. In conclusion, adding PRP lotion is superior to XONRID® gel in preventing and delaying ARD, reducing its severity, and improving patients’ quality of life. Future studies should evaluate the effectiveness of PRP lotion for ARD prevention in patients being treated for other cancer types, such as head and neck cancers.

## Funding

The study was supported by grants from Tri-Service General Hospital (TSGH-d-112071 and TSGH-d-113084) and National Defense Medical Center (MND-MAB-d-113060), and the Research Fund of Cardinal Tien Hospital (CTH112A-NDMC-2226).

## Conflicts of Interest

The author(s) have no conflicts of interest to declare.
